# Loss of LanC-like proteins delays post-injury regeneration of aging skeletal muscles

**DOI:** 10.64898/2026.05.15.725287

**Published:** 2026-06-15

**Authors:** Adriana Reyes-Ordoñez, Tianhui Hina Zhou, Tarun C. Rao, Pallob Barai, Wilfred A. van der Donk, Jie Chen

**Affiliations:** 1 Department of Cell & Developmental Biology, Carle Illinois College of Medicine, University of Illinois at Urbana-Champaign, Illinois, USA; 2 Department of Biochemistry, Carle Illinois College of Medicine, University of Illinois at Urbana-Champaign, Illinois, USA; 3 Department of Bioengineering, Carle Illinois College of Medicine, University of Illinois at Urbana-Champaign, Illinois, USA; 4 Department of Chemistry, Carle Illinois College of Medicine, University of Illinois at Urbana-Champaign, Illinois, USA; 5 Howard Hughes Medical Institute, Carle Illinois College of Medicine, University of Illinois at Urbana-Champaign, Illinois, USA; 6 Cancer Center at Illinois, Carle Illinois College of Medicine, University of Illinois at Urbana-Champaign, Illinois, USA; 7 Department of Biomedical and Translational Sciences, Carle Illinois College of Medicine, University of Illinois at Urbana-Champaign, Illinois, USA; 8 These authors contributed equally to the work

## Abstract

The adult skeletal muscle regenerates robustly upon injury, but this regenerative capacity rapidly declines with age. In this study, we identify the lanthionine synthetase C-Like (LanCL) proteins, mammalian homologs of the bacterial peptide cyclase LanC, as positive regulators of muscle regeneration in middle-aged mice. In a barium chloride-induced injury model, we found the protein levels of LanCL1 and LanCL2 to increase during an early phase of regeneration in middle-aged (12-month-old) but not young adult (4-month-old) mice. Utilizing a mouse line lacking all three LanCL proteins (LanCL triple KO or LTKO), we examined a potential role of LanCL in injury-induced muscle regeneration. Consistent with an age-dependent function of LanCL, we observed a delayed regeneration of the tibialis anterior (TA) muscle after injury, as reflected by reduced sizes of regenerating myofibers at day 7 after injury in middle-aged (but not young) LTKO compared to age-matched WT mice. Although the pool size of quiescent satellite cells (Pax7+) was comparable between 12-month-old LTKO and WT muscles without injury, the number of Pax7+ cells was significantly higher in regenerating LTKO muscles at day 5 after injury, accompanied by drastically decreased numbers of MyoD+ and MyoG+ cells, as well as increased numbers of proliferating cells. In addition, we detected elevated expression of pro inflammatory cytokines in regenerating LTKO muscles, while the number of macrophages was similar comparing LTKO and WT muscles. Taken together, our observations suggest that in aging muscles LanCLs are important for proper timing of inflammation resolution and regeneration upon injury.

## New & Noteworthy

Physiological roles of the mammalian homologs of bacterial LanC, LanCLs, are poorly understood. Our work uncovers a function of LanCLs in post-injury regeneration of aging skeletal muscles. Middle-aged LanCL triple KO mice displayed a delay in satellite cell differentiation and regenerative myofiber formation, as well as persistent inflammatory cytokine expression, suggesting that LanCLs may have an age-dependent role in modulating inflammation in the injured muscles to facilitate regeneration.

## INTRODUCTION

Lanthionine synthetase C-Like protein 1, 2, and 3 (LanCL1, 2, 3) are mammalian homologs of the bacterial peptide cyclase LanC, which is involved in the production of lanthipeptides, members of a natural product family called the ribosomally synthesized and post-translationally modified peptides (RiPPs) [1]. Lanthipeptides are characterized by lanthionine crosslinks formed from Ser and Cys residues. The Ser is dehydrated to the dehydroamino acid dehydroalanine (Dha), and LanC catalyzes the addition of Cys to the Dha to form the lanthionine crosslink [2]. However, LanCLs are not involved in lanthionine synthesis in mammals [3]. These evolutionarily conserved proteins were functionally uncharacterized until the early 2000s, but by now multiple independent studies have reported the involvement of LanCL1 and LanCL2 in redox homeostasis [4–11], protein damage control [12], cell survival [5, 7, 13], and immune responses [14–17]. Like LanC, LanCLs are zinc-binding proteins with evolutionarily conserved active site residues [18]. However, instead of catalyzing intramolecular ring formation through the addition of Cys to Dha, LanCLs catalyze C-glutathionylation of dehydroamino acids generated at protein phosphorylation sites, which may serve to protect the mammalian proteome from reactive electrophiles [12]. These findings lead to the speculation that LanCLs may be involved in protein damage control, especially during aging.

Both LanCL1 and LanCL2 are highly expressed in the brain, and LanCL1 has been reported to provide protection against chronic neurodegeneration [7]. LanCL1 has also been identified as a critical sensor and regulator of cellular glutathione (GSH) levels through negative regulation of Cystathionine β-Synthase (CBS) [11], making it an antioxidant protein that promotes neuron survival and cancer cell proliferation [5, 7, 8]. Such an antioxidant role was also observed for LanCL2 in the regulation of testicular redox homeostasis [8]. In addition, LanCL2 was reported to modulate immune response during host-pathogen interactions [14], and it has been suggested to be a potential therapeutic target in treating autoimmune and inflammatory diseases [15, 16, 19]. Moreover, LanCL2 was found to regulate lipid metabolism by promoting adipogenic differentiation through regulating PPARγ [20], and to promote cell survival by facilitating mammalian target of rapamycin complex 2 (mTORC2) phosphorylation of the Ser/Thr kinase AKT [13].

LanCL1 and LanCL2 are both expressed in skeletal muscles [21]. The adult skeletal muscle has a robust capacity to repair and regenerate upon injury, owing to the resident muscle stem cells (MuSCs) that can undergo activation, proliferation and differentiation to form new myofibers. This process requires the coordinated actions of multiple cell types, working in tandem with MuSC in the regenerative niche. For instance, macrophages play critical roles throughout the early stage of regeneration by clearing damaged tissues and promoting MuSC proliferation and differentiation. Redox signaling has long been recognized to play an essential role in skeletal muscle homeostasis and, in more recent years, has also been established as a strategic regulator of cellular and molecular processes required for efficient skeletal muscle regeneration [22]. Consistently, antioxidant enzymes are critically involved in muscle regeneration as they induce angiogenesis and reduce fibrosis [23]. Moreover, muscle repair/regeneration places a high demand on proteostasis. LanCL proteins’ involvement in antioxidation, inflammation, and protein damage control potentially put them at the intersection of key stages of muscle repair. Yet, a role of LanCL proteins in muscle regeneration has not been reported. In the current study we made use of a mouse line lacking expression of all three LanCL proteins [3] and observed that LanCL deletion resulted in delayed muscle regeneration upon acute injury. We further identified a dysregulated inflammatory response as a possible cause of the impaired regeneration.

## Materials and Methods

### Animals

All animal experiments in this study followed protocols approved by the Animal Care and Use Committee at the University of Illinois at Urbana-Champaign. LanCL1/2/3 triple knockout (LTKO) mice [3] and wild-type (WT) control mice were maintained on an FVB/NJ background (Jackson Laboratory, stock number 001800). Male and female LTKO and WT mice were used for experiments at 3 to 4 months of age (young), or 12 to 13 months (middle-aged; referred to as “aged”). Mice were housed up to 5 per cage. The cages were connected to an EcoFlo ventilation system (Allentown Inc.) in a specific-pathogen-free animal facility kept at 23 °C. All animals were maintained on a 12 h/12 h light/dark cycle with ad libitum access to water and normal chow.

### Skeletal muscle injury and regeneration

A barium chloride (BaCl_2_) injury-induced muscle regeneration model was used as previously described [24]. Briefly, the mouse was anesthetized by isoflurane in an induction chamber (2–3%) and then maintained under anesthesia via a nosecone (1.5–2%) using a precision vaporizer and an active scavenger system. Tibialis anterior (TA) muscles were then injected with 50 μL of 1.2% (w/v) BaCl_2_ (Sigma, Cat. #202738) dissolved in saline (injured) or just saline (uninjured control). At 5, 7, or 14 days after injection, the injected TA muscles were collected from mice euthanized by cervical dislocation under anesthesia. The isolated muscles were immediately embedded in tissue freezing medium TBS (VWR, Cat. #15148–031) or snap-frozen in liquid nitrogen-cooled 2-methyl-butane (Fisher Scientific, Cat. #AC126470010). The samples were stored at −80 °C until further processing. Samples were excluded from analysis if it was determined that the extent of muscle injury was drastically less than expected (likely due to mis-injection).

### CSA measurement

Sections of 10-μm thickness of the TA muscle mid-belly were obtained using a cryostat (Microm HM550; ThermoFisher Scientific) at −20 °C and placed on charged slides, followed by staining with Hematoxylin (Sigma-Aldrich, Cat. #GHS316) and Eosin (Sigma-Aldrich, Cat. #HT110116) (H&E). H&E images were acquired using a microscope (DMI 4000B; Leica) with a 20× dry objective (Fluotar, numerical aperture 0.4; Leica) and a camera (RETIGA EXi; QImaging) controlled by the Q-Capture Pro51 software (QImaging). Bright-field images were captured at 12-bit at room temperature (RT) and processed using ImageJ (NIH), where the cross-sectional area (CSA) of centrally nucleated regenerating myofibers was measured. A minimum area of 1,480,000 μm^2^ at the center of the regenerating region of each TA muscle was analyzed for CSA. Investigators were blinded to sample identification during the procedures.

### Immunofluorescence imaging and analysis

For immunofluorescence, 10-μm muscle sections were rehydrated with phosphate-buffered saline (PBS), fixed with 3.7% formaldehyde (ThermoFisher Scientific, Cat. #F79–1) for 10 min, permeabilized using 0.1% Triton X-100 (ThermoFisher Scientific, Cat. #BP151) for 5 min, and blocked with 5% BSA (Sigma-Aldrich, Cat. #A7906) and 5% normal goat serum (Jackson ImmunoResearch, Cat. #005-000-001) for 60 min, all at RT. Incubation with primary antibodies was performed overnight at 4 °C. After washing with PBS + 1% BSA, the samples were incubated with Alexa fluor–conjugated secondary antibodies and 1 μg/ml of DAPI (ThermoFisher Scientific, Cat. #P162247) for 60 min at RT. For F4/80 staining, the samples were permeabilized after primary antibody incubation and washing. Fluorescent images were acquired with the same microscope setup described above, captured as 8-bit images at RT, and processed in ImageJ (NIH). Images were pseudo-colored and adjusted, when necessary, using identical parameters for all samples within the same experiment. A minimum area of 1,480,000 μm^2^ was scored for the number of fluorescent cells. For F4/80 analysis, the corrected total cell fluorescence (CTCF) was quantified using ImageJ. All antibodies used in this study are listed in Table S1. Investigators were blinded to sample identification during the procedures.

### mRNA extraction and quantitative real-time PCR

Ice-cold TRI reagent (Sigma-Aldrich, Cat. #T9424) was added to frozen muscles and homogenized using a hand-held homogenizer (ThermoFisher Scientific), followed by total mRNA extraction according to the manufacturer recommendations. mRNA concentrations were measured using a NanoDrop ND-2000C (ThermoFisher Scientific) and 1 μg of the total mRNA was reverse-transcribed using qScript cDNA Synthesis Kit (Quanta Bioscience, Cat. # 95048). Quantitative polymerase chain reaction (qPCR) was performed with SYBR green using a StepOnePlus Real-Time PCR System (Applied Biosystems). The qPCR reactions were run in triplicate for each sample. The qPCR primers used in this study are listed in Table S2. Data were processed using the 2^−ΔΔCt^ method [25]. Investigators were blinded to sample identification during the procedures.

### Protein extraction and western blotting

Frozen muscles were homogenized with a hand-held homogenizer (ThermoFisher Scientific) in ice-cold lysis buffer containing 20 mM Tris-HCl (pH 7.4), 25 mM NaF, 0.1 mM Na_3_VO_4_, 25 mM b-glycerolphosphate, 2 mM EDTA, 2 mM EGTA, 0.3% Triton X-100 and 1x protease inhibitor cocktail (Sigma-Aldrich, Cat. #P8340). The homogenates were pre-cleared by centrifugation at 16,200xg for 10 min at 4 °C and protein concentrations were determined using Bradford protein dye (BioRad, Cat. #5000006). Protein samples (15 μg per sample) were separated by sodium dodecyl sulfate-polyacrylamide gel electrophoresis (SDS-PAGE) and transferred to polyvinylidene fluoride (PVDF) membranes (ThermoFisher Scientific, Cat. #PI88520). After blocking in 5% non-fat dry milk in PBS with 0.1% (v/v) Tween 20 (PBST), membranes were incubated with the primary antibody overnight at 4 °C, followed by washing with PBST and incubation with horseradish peroxidase-conjugated secondary antibody for 30 min at RT. All antibodies and their dilutions used are listed in Supplemental Table S1. Chemiluminescent signals were developed using SuperSignal Western Pico PLUS Chemiluminescent Substrate (ThermoFisher Scientific, Cat. #34580) and captured on an iBright CL1000 imaging system (ThermoFisher Scientific). Western blot images were quantified by densitometry in ImageJ (NIH) to obtain relative protein levels, and normalized to GAPDH as the control.

### Single-cell RNA sequencing data analysis

We analyzed target gene expression using publicly available mouse skeletal muscle single-cell RNA sequencing (scRNA-seq) datasets. Raw scRNA-seq data from multiple published datasets were obtained, covering early regeneration time points (0, 2, 5, and 7 days after injury) from both young adult (4–7 months) and old (20 months) mice [26, 27]. GEO accession information for datasets used can be found in Table S3. Processing and analysis of the raw sequencing datasets were performed as previously described [28]. Briefly, after quality control, normalization, dimensionality reduction, integration, clustering, and cell type annotation, the expression of *Lancl1* and *Lancl2* were quantified across defined cell populations and over regeneration time points. Pseudo-bulk differential expression analysis was performed on scRNA-seq data by aggregating raw gene expression levels using the AggregateExpression function in Seurat across cells within each cell type for each biological replicate. Differential expression analysis between different regeneration timepoints was subsequently conducted using DESeq2 on the aggregated counts.

### Statistical analysis

The exact sample size for each experiment is described in figure legends. All quantitative data are presented as mean ± SEM. Unpaired two-tailed student’s *t* test was performed in Excel to assess statistical significance of differences between two groups, and *p* < 0.05 was considered statistically significant.

## RESULTS

### Expression of LanCL1 and LanCL2 proteins in regenerating skeletal muscles

**Fig. 1.**
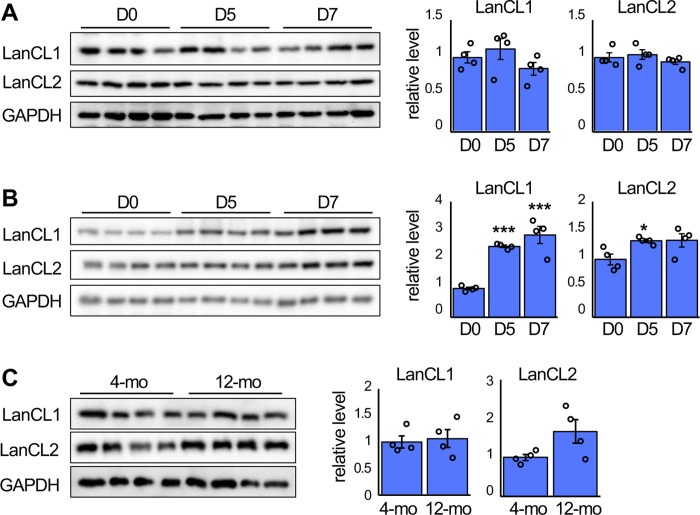
LanCL1 and LanCL2 protein expression during muscle regeneration. (**A-B**) TA muscles of 4-month-old (A) or 12-month-old (B) mice were injected with BaCl_2_. At D5AI and D7AI the injected muscles were isolated and subjected to homogenization and analysis by western blotting. Saline injection served as D0 control. Each experimental group contained 4 animals. Western blots were quantified by densitometry, and GAPDH was used as a reference for relative protein expression levels. Quantification data are presented as mean ± SEM, with individual data points shown. (**C**) TA muscles of 4-month-old or 12-month-old mice without injury were isolated and analyzed by western blotting as described above. Quantification data are presented as mean ± SEM, with individual data points shown. Two-tailed Student’s t test was performed to analyze statistical significance for comparison between D0 and D5 or D7 (for A and B) or between 4-mo and 12-mo (for C). *p < 0.05; **p < 0.01; ***p < 0.001.

LanCL1 and LanCL2 are broadly expressed in most mammalian tissues including the skeletal muscle, whereas LanCL3 expression is very low at the transcript level in all tissues and information on its protein expression is scarce due to the lack of optimal reagents (The Human Protein Atlas [21]). To probe a potential involvement of LanCL1 and LanCL2 in injury-induced muscle regeneration, we started by examining the expression of these proteins in regenerating muscles of wild type mice post-injury. The tibialis anterior (TA) muscle was injured by barium chloride (BaCl_2_) injection and isolated on day 5 or 7 after injury (D5AI or D7AI), followed by protein extraction and western blotting analysis. Uninjured muscles were also collected for analysis (D0). In young adult mice at 3–4 months of age, the expression levels of LanCL1 and LanCL2 did not change markedly comparing regenerating muscles at D5AI or D7AI to uninjured muscles (D0) (Fig. 1A). However, at 12 months of age (middle-aged), expression of both LanCL1 and LanCL2 increased upon injury (Fig. 1B). When comparing young and middle-aged muscles without injury, we observed no difference in the expression level of LanCL1 or LanCL2 (Fig. 1C). These observations imply that LanCL1/2 may have a unique function in injury response of aging muscles.

**Fig. 2.**
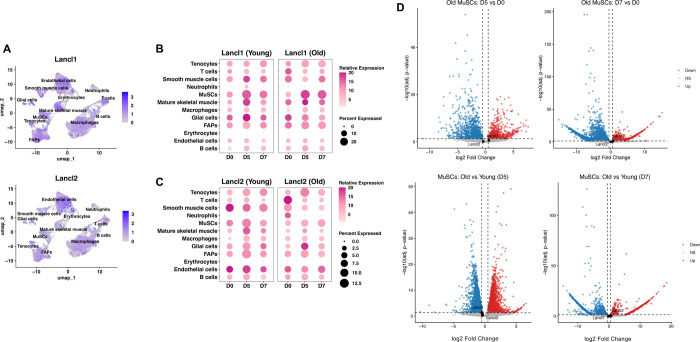
ScRNA-seq analysis of expression of *Lancl1* and *Lancl2* during muscle regeneration. scRNA-seq datasets of mouse TA muscles at D0, D2, D5, and D7AI were integrated and analyzed. (**A**) Single-cell atlases of 170,088 cells combining the timepoints are shown. Data are presented in UMAP to visualize the transcript levels of Lancl1 and Lancl2 in various cell clusters. (**B**) Dot-plots show percentage of cells expressing Lancl1 and relative expression level in various cell clusters of young (4–7 months) and old (20 months) mouse TA muscle at D0, D5, and D7AI. (**C**) Similar to (B) for expression of Lancl2. (**D**) Pseudo-bulk analysis was performed with the scRNA-seq data to identify global transcriptional changes in young and old MuSCs during regeneration. Volcano plots show differentially expressed genes comparing D5AI or D7AI with D0AI in old MuSCs (upper) or comparing old and young MuSCs at D5 and D7AI (lower). Lancl1 and Lancl2 are not differentially expressed.

Next, we interrogated the vast single-cell RNA sequencing (scRNA-seq) datasets publicly available [29] to gain cell type-specific insights into the mRNA expression of Lancl genes in regenerating muscles. We performed integration of scRNA-seq data for uninjured (D0) and regenerating TA muscles at D5AI and D7AI for young (4–7 months) and aged (20 months) mice, and analyzed the transcripts of *Lancl1* and *Lancl2* in the integrated datasets. As expected, *Lancl3* mRNA was barely detectable in this dataset. As shown in Fig. 2A, both *Lancl1* and *Lancl2* genes were widely expressed across all cell types in muscles when combining data from all time points of regeneration. In both young and aged muscles, muscle stem cells (MuSCs) displayed increased levels of *Lancl1* mRNA expression on D5AI and D7AI compared to D0 (Fig. 2B). *Lancl2* mRNA levels also appeared to increase during regeneration of both young and aged muscles, albeit to lesser degrees (Fig. 2C). Both *Lancl1* and *Lancl2* expression, in both young and old muscles, were relatively modest in macrophages, the major type of immune cells involved in muscle regeneration (Fig. 2B–C). While the single-cell analysis revealed trends of expression, conclusions about expression level change cannot be drawn from this type of analysis. To gain insights into gene expression across biological replicates from the existing scRNA-seq datasets, we performed pseudo-bulk gene expression analysis for MuSCs. When comparing D5AI and D7AI to D0, no statistically significant change was found for *Lancl1* or *Lancl2* in aged MuSCs (Fig. 2D). There was also no significant difference in *Lancl1* or *Lancl2* expression comparing young and aged MuSCs at D5AI or D7AI (Fig. 2D). In conclusion, the mRNA expression patterns derived from scRNA-seq data are distinct from those of protein expression (Fig. 1), suggesting that LanCL1 and LanCL2 expression during muscle regeneration may be under post-transcriptional regulation.

### Loss of LanCL proteins leads to delayed muscle regeneration

**Fig. 3.**
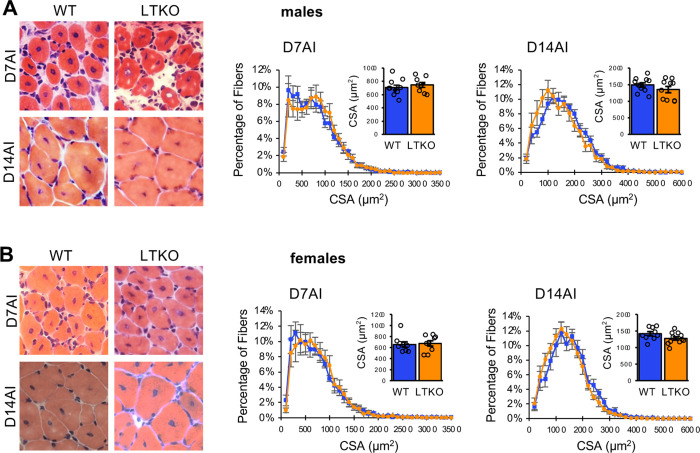
Normal muscle regeneration in young adult LTKO mice. TA muscles of male (**A**) and female (**B**) 4-month-old WT and LTKO mice were subjected to BaCl_2_ injury and isolated at D7AI and D14AI. Cryosections of regenerating muscles were stained with H&E, and cross-sectional area (CSA) of centrally nucleated myofibers was measured. Representative H&E images are shown. Myofiber size distribution (mean ± SEM) and average CSA (mean ± SEM) are shown. Blue: WT; orange: LTKO. Two-tailed Student’s t test was performed to analyze statistical significance. No significant difference was found between WT and LTKO,

**Fig. 4.**
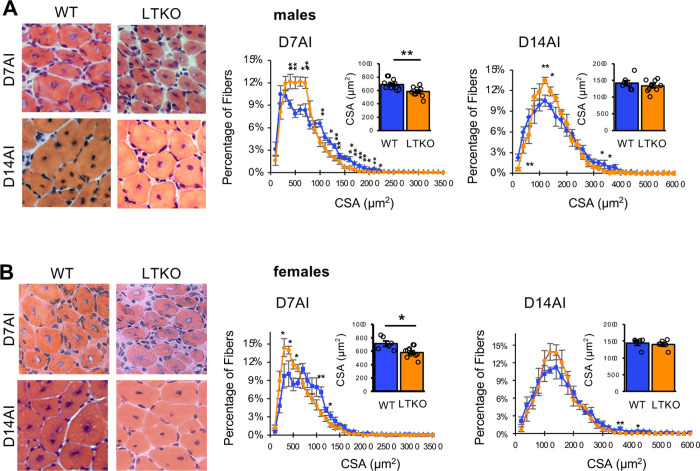
Delayed muscle regeneration in middle-aged LTKO mice. TA muscles of (**A**) young (4-month-old) and (**B**) middle-aged (12-month-old) WT and LTKO male (left panels) and female (right panels) mice were subjected to BaCl_2_ injury and isolated at D7AI and D14AI. Cryosections of regenerating muscles were stained with H&E, and cross-sectional area (CSA) of centrally nucleated myofibers was measured. Representative H&E images are shown. Myofiber size distribution (mean ± SEM) and average CSA (mean ± SEM) are shown. Blue: WT; orange: LTKO. Two-tailed Student’s t test was performed to analyze statistical significance. *p < 0.05; **p < 0.01; ***p < 0.001.

The protein expression patterns of LanCL1 and LanCL2 described above implicated these proteins in a potential function during muscle repair in aging. To investigate the role of LanCL proteins, we took advantage of a LanCL triple knockout mouse line with all three Lancl genes disrupted and protein expression abolished (henceforth referred to as LTKO) [3]. The LTKO mice do not display any gross phenotype [3]. BaCl_2_ injection was performed to injure TA muscles, followed by isolation of the injured muscles at D7AI and D14AI, and quantification of the average cross-sectional area (CSA) of regenerating myofibers to assess the degree of regeneration. In young adult mice, the average regenerating myofiber CSA or the size distribution of CSA was not significantly different comparing WT and LTKO muscles at D7AI or D14AI in either male (Fig. 3A) or female mice (Fig. 3B). At 12 months of age, LTKO animals exhibited reduced average CSA compared with WT at D7AI and a left shift of the CSA size distribution, observed in both male (Fig. 4A) and female (Fig. 4B) mice. Statistically significant difference was no longer found in average CSA by D14AI (Fig. 4A&B). These observations indicate that loss of the LanCL proteins resulted in an age-dependent delay in muscle regeneration. This transient impairment observed in older LTKO mice suggests a possible role for the LanCL proteins in an early stage of regeneration, such as satellite cell activation or differentiation, or inflammatory responses.

### Satellite cell differentiation is delayed in LTKO muscles

**Fig. 5.**
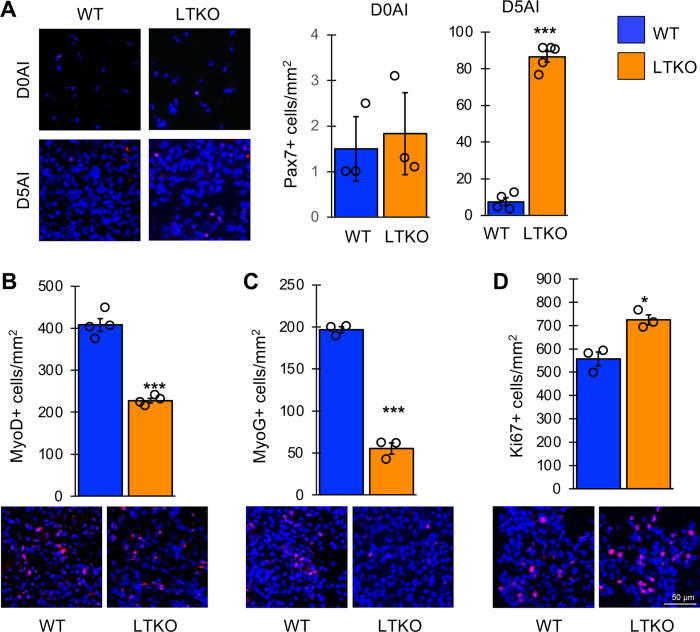
Delayed satellite cell differentiation in regenerating muscles of middle-aged LTKO mice. (**A**) TA muscles of 12-month-old WT and LTKO mice were subjected to BaCl_2_ injury or saline injection (D0AI) and isolated at D5AI. Cryosections of muscles were immunostained for Pax7, and fluorescently positive cells were counted. (**B-D**) TA muscles of 12-month-old WT and LTKO mice injured by BaCl_2_ were isolated at D5AI and subjected to immunostaining for MyoD (B), MyoG (C), or Ki-67 (D), and fluorescently positive cells were counted. Representative images are shown, and quantification data are presented as average number of positive cells per field ± SEM, with individual data points shown. Blue bars: WT; orange bars: LTKO. Two-tailed Student’s t test was performed to analyze statistical significance for comparison between WT and LTKO. *p < 0.05; **p < 0.01; ***p < 0.001.

To investigate whether satellite cell behavior was affected by loss of LanCL proteins in 12-month-old muscles, we performed immunohistochemical analysis of TA muscle cross sections before injury (D0AI) and during regeneration at D5AI. Pax7 is known to be expressed in quiescent as well as activated satellite cells, but not in differentiating myocytes. As shown in Fig. 5A the number of Pax7+ cells per cross sectional area was comparable between WT and LTKO muscles prior to injury, suggesting that loss of LanCL proteins did not affect the quiescent muscle stem cell pool. On the other hand, at D5AI we observed a drastically higher population of Pax7+ cells in LTKO (Fig. 5A). Compared to the WT, LTKO muscles also contained significantly lower numbers of MyoD+ cells (Fig. 5B) and cells expressing MyoG, a marker of myogenic differentiation (Fig. 5C). On the other hand, the number of proliferating cells, as marked by Ki-67, was significantly higher in LTKO muscles (Fig. 5D). Taken together, these observations suggest that LTKO satellite cells may be stalled in the proliferation stage and delayed in their entry into differentiation, which is consistent with the observed delay in new myofiber formation (Fig. 4).

### Loss of LanCL proteins delays resolution of inflammation during regeneration

The initial pro-inflammatory response to injury as well as timely resolution of inflammation is critical for injury-induced muscle regeneration. Following acute skeletal muscle injury, infiltrating immune cells, mainly macrophages, adopt an initial pro-inflammatory, debris-clearing, and pro-proliferative program in the first 1–2 days post-injury. After that, the macrophages undergo a transition into a reparative role aiming to support MuSCs differentiation, growth and remodeling. This transition is reported to begin around day 2 and be completed around day 4–5 post injury, following which macrophages decline in number but play a supportive role in myoblast differentiation and fusion as well as contributing to vascular and extracellular matrix remodeling [30–32]. To understand how the loss of LanCL proteins could lead to delayed myogenic differentiation and regeneration in aged mice, we set out to investigate the inflammatory response and the shift of gene expression programs from pro-inflammation to pro-regeneration in WT versus LTKO muscles. We performed qRT-PCR to quantify the mRNA levels of multiple pro- and anti-inflammatory markers in injured TA muscle at D5AI, a key time point in the inflammatory-to-reparative transition.

**Fig. 6.**
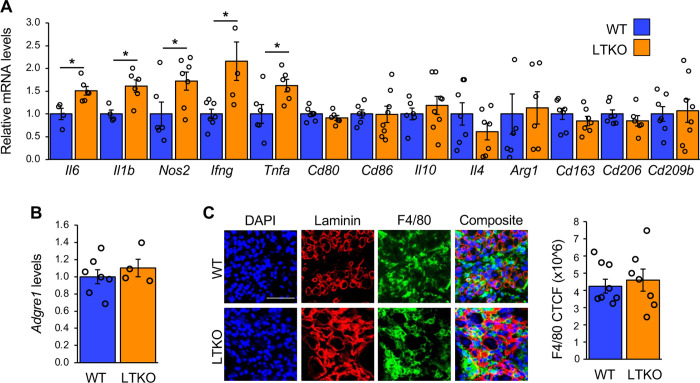
Delayed resolution of inflammation in LTKO muscles after injury. (**A-B**) TA muscles of 12-month-old WT and LTKO mice were subjected to BaCl_2_ injury and isolated at D5AI, followed by RNA extraction and qPCR analysis of relative levels of mRNA expression. Data are presented as mean ± SEM with individual data points shown. (**C**) The same muscles in (A-B) were cryosectioned and immunostained with anti-F4/80 and anti-Laminin. Representative images are shown. F4/80 fluorescent signals were quantified as corrected total cell fluorescence (CTCF). Data are presented as mean ± SEM. Two-tailed Student’s t test was performed to analyze statistical significance. *p < 0.05.

We examined the expression of the following mRNAs: markers associated with pro-inflammatory activity (M1-like) macrophage activation (*Il6, Il1b, Nos2, Tnfa, Ifng*, *Cd80* and *Cd86*), markers typically associated with anti-inflammatory transition (*Il10 and Il4)*, and markers associated with reparative (M2-like) macrophages *(Arg1, Cd163, Cd206* and *Cd209b).* Comparing 12-month-old LTKO and WT muscles, we observed significantly elevated levels of *Il6, Il1b, Ifng, Tnfα,* and *Nos2* (Fig. 6A), suggesting a prolonged pro-inflammatory state, which might have resulted in delayed satellite cell differentiation. No difference was found in the expression of Adgre1, a pan-macrophage marker, between WT and LTKO (Fig. 6B). The number of F4/80+ cells (all macrophages) in TA muscles was also comparable between the two genotypes (Fig. 6C).

## DISCUSSION

In this study, we identified a key role for LanCL proteins in the dynamic process of post-injury muscle regeneration upon aging. Disruption of all three Lancl genes in mice led to a transient yet significant delay of muscle regeneration in middle-aged but not young adult mice. This impairment does not appear to be due to any change in the quiescent MuSC pool size or its activation. Rather, a delay in the transition from proliferation to differentiation of MuSCs, accompanied by prolonged pro-inflammatory cytokine expression, may have contributed to the observed regeneration phenotype. This aging-dependent phenotype was consistent with the observation that LanCL1 and LanCL2 protein expression were upregulated during regeneration only in the older mice, suggesting that these proteins may have an important function in the aging muscle. Whether the two LanCL proteins play redundant or unique roles is a question that warrants future studies with single KOs. A role of LanCL3 also cannot be definitively ruled out until further investigation.

A key aspect not addressed by our current study is the specific cell type in which LanCL proteins exert their regulatory functions in muscle regeneration during aging. The observed delay in MuSC transition from proliferation to differentiation in LTKO regenerating muscles could reflect a cell-autonomous function of LanCL in the stem cells. However, the prolonged expression of pro-inflammatory cytokines in aged LTKO regenerating muscles would be consistent with a function of LanCLs in immune cells, which impacts MuSC behaviors. A potential involvement of LanCL2 in inflammation has been implicated by its connection to autoimmune conditions and inflammatory diseases [19]. Although mRNA levels of Lancl1 and Lancl2 are relatively low in macrophages throughout the regeneration time course and regardless of age (Fig. 2), information on cell type-specific LanCL protein expression is not available, and a potential LanCL function in macrophages remains a possibility. A third possibility also exists, considering the growing recognition of bi-directional regulation between MuSCs and immune cells [28, 31, 33, 34]. While the macrophage is long considered a key contributor to regulating MuSC functions, MuSCs are emerging regulators of immune cells during muscle regeneration. Therefore, it is plausible that LanCL proteins may act in MuSCs to exert immunomodulating functions, which in turn regulates the progression of MuSCs through early stages of regeneration. These possibilities are not mutually exclusive, and future investigations probing the various models may prove fruitful.

An overall well-controlled and timed redox state is another essential component of skeletal muscle regeneration, not only in MuSCs but in all cell types present in the muscle, and this redox balance is progressively destabilized during aging [22, 35]. Reactive oxygen species (ROS) generated in injured skeletal muscles impact inflammation and MuSC activation, proliferation, differentiation and self-renewal [22]. LanCL1 has been reported to mitigate oxidative stress and preserve mitochondrial function in neurons [5, 9], as well as protecting cancer cells from oxidative stress-induced apoptosis by suppressing JNK signaling [8]. Chronic oxidative stress, weaker antioxidant buffering, and altered nitric oxide signaling are all contributing factors to a delayed shift from inflammation to myogenesis during regeneration in aging muscles [23, 35, 36]. Glutathione (GSH) is the most abundant small antioxidant molecule in skeletal muscles, and aged MuSCs with lower GSH levels are found to have reduced regenerative capacity [37]. LanCL1 and LanCL2 use glutathione as a substrate for post translational C-glutathionylation of various mammalian proteins [12, 38]. Structural studies have shown that GSH binds to LanCL1’s active site [18] and LanCL1 has affinity for the signaling protein Eps8, linking LanCL1’s redox regulatory function to cell signaling in the nervous system [39]. LanCL1’s antioxidant function is further supported by developmental studies showing that its expression is necessary for neuronal survival under ROS stress [5], and LanCL1 overexpression protects mice with misfolded SOD1G93A from increased oxidative damage [7]. It is possible that LanCL deficiency exacerbated the pre-existing redox imbalance in aging muscles due to impaired GSH buffering and compromised antioxidant response, resulting in delayed regeneration.

The function of LanCL proteins in regeneration may also involve their recently discovered role as enzymes that protect the mammalian proteome from accumulation of dehydroamino acid (DHAA) containing proteins. DHAAs have been well documented to accumulate in aging tissues, causing loss of protein function, irreversible cross-linking and insolubility [12, 40, 41]. Eliminating damaged proteins is fundamental to maintaining a healthy proteome in aging, without which cellular signaling as well as enzymatic reactions that are essential for post injury muscle repair and regeneration would not proceed normally. DHAA has mainly been reported in the aging lens and brain associated with Alzheimer’s disease [40, 42], and its prevalence and relevance in skeletal muscle has not been studied, possibly due to low abundance and fast protein turnover. Our findings provide the first evidence for LanCL proteins as regulators of the finely tuned process of muscle regeneration, particularly in the context of aging. Determining whether LanCLs play a critical role in mitigating aging-induced protein damage in the regenerating muscle represents an exciting area for future studies.

## Supplementary Material

Supplement 1
