# Supplementary material for: Loss of LanC-like proteins delays post-injury regeneration of aging skeletal muscles
Source: bioRxiv. 2026 Jun 15:2026.05.15.725287. Preprint. [Version 2] doi: 10.64898/2026.05.15.725287 (PMC13307945; doi:10.64898/2026.05.15.725287)
Supplement: Supplement 1 [file media-1.pdf]

Supplemental Table S1: List of Antibodies used

Type	ID	Source	Catalog #	RRID	Dilution Used
Primary Antibodies	Rabbit polyclonal anti-Pax7	Aviva Systems Biology	ARP32742_P050	AB_387565	1:800
	Myogenin (E9A1S) Rabbit Monoclonal Antibody	Cell Signaling Technology ®	43098	AB_3717698	1:100
	MYOD1 Polyclonal antibody	Proteintech®	189043-I-AP	AB_10603467	1:500
	Ki-67 (D3B5) Rabbit monoclonal	Cell Signaling	12202	AB_2620142	1:600
	Rat anti Mouse F4/80	BIO-RAD	MCA497	AB_2098196	1:200
	LANCL1 Monoclonal Antibody	Proteintech®	68160-1-Ig	AB_2923679	1:1000
	Anti-LANCL2	Jie Chen Lab (UIUC); Zeng M. et al, 2014	n/a	N/A	1:1000
	GAPDH Polyclonal antibody	Proteintech®	10494-1-AP	AB_2263076	1:5000
Secondary Antibodies	Alexa Fluor® 488 AffiniPure® F(ab')$_2$ Fragment Goat Anti-Rabbit IgG (H+L)	Jackson Immunoresearch Inc	111-546-003	AB_2338053	1:500
	Alexa Fluor® 488 conjugated Donkey Anti Rat IgG	Jackson Immunoresearch Inc	712-545-150	AB_2340683	1:500
	Alexa Fluor® 594 AffiniPure® F(ab')$_2$ Fragment Goat Anti-Rabbit IgG (H+L)	Jackson Immunoresearch Inc	111-586-003	AB_2338066	1:500
	Peroxidase AffiniPure® Goat Anti-Rabbit IgG (H+L)	Jackson Immunoresearch Inc	111-035-003	AB_2313567	1:2000
	Peroxidase AffiniPure® Goat Anti-Mouse IgG (H+L)	Jackson Immunoresearch Inc	115-035-003	AB_10015289	1:2000

Supplemental Table S2: List of primers used for qPCR analysis

Gene	Forward 5'-3'	Reverse 5'-3'
Il-6	TGTTCTCTGGGAAATCGTGGAA	GCAAGTGCATCATCGTTGTTCA
Il1b	GAAAGCTCTCCACCTCAATG	GCCGTCTTTCATTACACAGG
Nos2	CTTGGAGCGAGTTGTGGATT	CTCTTGTCTTTGACCCAGTAGC
Infγ	GGCCATCAGCAACAACATAAG	GTTGACCTCAAACTTGGCAATAC
CD-80	TGCTGCTGATTCGTCTTTCAC	GAGGAGAGTTGTAACGGCAAG
Tnfα	GCCTCTTCTCATTCCTGCTTG	CTGATGAGAGGGAGGCCATT
CD-80	CATGGGCTTGGCAATCCTTA	AAATGGGCACGGCAGATATG
Il-10	TTGAATTCCCTGGGTGAGAAG	TCCACTGCCTTGCTCTTATTT
Il-4	GAATGTACCAGGAGCCATATCC	ACGTTTGGCACATCCATCT
Arg1	CTCCAAGCCAAAGTCCTTAGAG	GGAGCTGTCATTAGGGACATCA
Cd163	AATCACATCATGGCACAGGTCACC	TCGTCGCTTCAGAGTCCACAGG
Cd206	CTTCGGGCCTTTGGAATAAT	TAGAAGAGCCCTTGGGTTGA
CD209b	GGCTAAAGGACCAACCTGGATG	CTCACCGATGTTGTTAGGCTCC
Adgre1	GGATGTACAGATGGGGGATG	CATAAGCTGGGCAAGTGGTA
Hprt	TCCAGCAGGTCAGCAAAGAAC	ATGGACTGATTATGGACAGGACTG

Supplemental Table S3: Accession numbers for publicly available scRNA seq used for analysis

Sample ID	Age	Age (Months)	Citation	GEO Accession	GEO Accession Sample ID
Yng_D0_A	Young	7 mo	De Micheli et al., PMID: 32160558	GSE143437	D0_A
Yng_D0_B	Young	7 mo	De Micheli et al., PMID: 32160558	GSE143437	D0_B
Yng_D0_C	Young	5 mo	De Micheli et al., PMID: 32160558	GSE143437	D0_Cv3
Yng_D2_A	Young	4 mo	De Micheli et al., PMID: 32160558	GSE143437	D2_C
Yng_D2_B	Young	4 mo	De Micheli et al., PMID: 32160558	GSE143437	D2_D
Yng_D2_C	Young	7 mo	Mckellar et al., PMID: 34773081	GSE159500	D2_Ev3
Yng_D5_A	Young	4 mo	De Micheli et al., PMID: 32160558	GSE143437	D5_A
Yng_D5_B	Young	4 mo	De Micheli et al., PMID: 32160558	GSE143437	D5_B
Yng_D5_C	Young	4 mo	De Micheli et al., PMID: 32160558	GSE143437	D5_C
Yng_D7_A	Young	4 mo	De Micheli et al., PMID: 32160558	GSE143437	D7_C
Yng_D7_B	Young	4 mo	De Micheli et al., PMID: 32160558	GSE143437	D7_D
Old_D0_A	Old	20 mo	Mckellar et al., PMID: 34773081	GSE162172	Old_D0_A
Old_D0_B	Old	20 mo	Mckellar et al., PMID: 34773081	GSE162172	Old_D0_B
Old_D0_C	Old	20 mo	Mckellar et al., PMID: 34773081	GSE162172	Old_D0_C
Old_D0_D	Old	20 mo	Mckellar et al., PMID: 34773081	GSE162172	Old_D0_D
Old_D2_A	Old	20 mo	Mckellar et al., PMID: 34773081	GSE162172	Old_D2_A
Old_D2_B	Old	20 mo	Mckellar et al., PMID: 34773081	GSE162172	Old_D2_B
Old_D2_C	Old	20 mo	Mckellar et al., PMID: 34773081	GSE162172	Old_D2_C
Old_D5_A	Old	20 mo	Mckellar et al., PMID: 34773081	GSE162172	Old_D5_A
Old_D5_B	Old	20 mo	Mckellar et al., PMID: 34773081	GSE162172	Old_D5_B
Old_D5_C	Old	20 mo	Mckellar et al., PMID: 34773081	GSE162172	Old_D5_C
Old_D7_A	Old	20 mo	Mckellar et al., PMID: 34773081	GSE162172	Old_D7_A
Old_D7_B	Old	20 mo	Mckellar et al., PMID: 34773081	GSE162172	Old_D7_B
Old_D7_C	Old	20 mo	Mckellar et al., PMID: 34773081	GSE162172	Old_D7_C
